# Effective coverage of essential inpatient care for small and sick newborns in a high mortality urban setting: a cross-sectional study in Nairobi City County, Kenya

**DOI:** 10.1186/s12916-018-1056-0

**Published:** 2018-05-22

**Authors:** Georgina A. V. Murphy, David Gathara, Jacintah Mwachiro, Nancy Abuya, Jalemba Aluvaala, Mike English, on behalf of the Health Services that Deliver for Newborns Expert Group, Sam Ochola, Robert Ayisi, Aggrey Wasunna, Fred Were, Catherine Mutinda, Beth Maina, Cecilia Mutiso, David Githanga, David Kimutai, Rachel Musoke, Roseline Ochieng, William Macharia, Rachel Nyamai

**Affiliations:** 1 Centre for Tropical Medicine and Global Health, Nuffield Department of Medicine, University of Oxford, Oxford, UK; 2 Kenya Medical Research Institute - Wellcome Trust Research Programme, Nairobi, Kenya; 3 Nairobi City County Government, Nairobi, Kenya

**Keywords:** Newborn health, Neonatal care, Effective coverage, Inpatient newborn services, Quality of care, Paediatrics, Health services research, Kenya, Africa

## Abstract

**Background:**

Effective coverage requires that those in need can access skilled care supported by adequate resources. There are, however, few studies of effective coverage of facility-based neonatal care in low-income settings, despite the recognition that improving newborn survival is a global priority.

**Methods:**

We used a detailed retrospective review of medical records for neonatal admissions to public, private not-for-profit (mission) and private-for-profit (private) sector facilities providing 24×7 inpatient neonatal care in Nairobi City County to estimate the proportion of small and sick newborns receiving nationally recommended care across six process domains. We used our findings to explore the relationship between facility measures of structure and process and estimate effective coverage.

**Results:**

Of 33 eligible facilities, 28 (four public, six mission and 18 private), providing an estimated 98.7% of inpatient neonatal care in the county, agreed to partake. Data from 1184 admission episodes were collected. Overall performance was lowest (weighted mean score 0.35 [95% confidence interval or CI: 0.22–0.48] out of 1) for correct prescription of fluid and feed volumes and best (0.86 [95% CI: 0.80–0.93]) for documentation of demographic characteristics. Doses of gentamicin, when prescribed, were at least 20% higher than recommended in 11.7% cases. Larger (often public) facilities tended to have higher process and structural quality scores compared with smaller, predominantly private, facilities. We estimate effective coverage to be 25% (estimate range: 21–31%). These newborns received high-quality inpatient care, while almost half (44.5%) of newborns needed care but did not receive it and a further 30.4% of newborns received an inadequate service.

**Conclusions:**

Failure to receive services and gaps in quality of care both contribute to a shortfall in effective coverage in Nairobi City County. Three-quarters of small and sick newborns do not have access to high-quality facility-based care. Substantial improvements in effective coverage will be required to tackle high neonatal mortality in this urban setting with high levels of poverty.

**Electronic supplementary material:**

The online version of this article (10.1186/s12916-018-1056-0) contains supplementary material, which is available to authorized users.

## Background

The provision of good quality inpatient care for small and sick newborns is crucial if efforts to achieve the sustainable development goal 3 target to reduce neonatal mortality to 12 per 1000 live births or lower is to be achieved [1]. To date, progress in addressing neonatal mortality has been slow and insufficient emphasis has been placed on the importance of quality health care for small and sick newborns [2–5].

The main causes of neonatal mortality are intrapartum-related neonatal encephalopathy, prematurity and neonatal sepsis [3], which together accounted for more than 75% of the observed mortality in Kenya in 2014 [6]. Much of the mortality and long-term morbidity associated with these conditions is preventable with cost-effective interventions and the provision of universal access to basic but high-quality health services [7, 8], including interventions provided to inpatients, such as intravenous antibiotics for sepsis, assisted feeding for premature newborns and oxygen for respiratory distress syndrome [9, 10]. Yet, many sick and small (preterm and small for gestational age) newborns are not receiving appropriate inpatient neonatal services, and those who are may be treated in suboptimal conditions [4, 11–13]. Failures in the delivery of best care may reduce the effectiveness of available services or even cause harm [14].

Despite having a high density of health facilities and 89% of women delivering in a health facility, Nairobi City County has the highest neonatal mortality of all regions in Kenya [15, 16]. Gaps in provision and access to effective services for neonatal care in Kenya have previously been highlighted in the public sector [11–13], yet little has been described about the non-public sector.

To address this, the Nairobi Newborn Study aimed to examine the provision, access and quality of inpatient neonatal care in Nairobi City County—a population of approximately four million, 60–70% of whom are slum dwellers [17]. Previous reports deal with the need for health services [18] and characterise structural aspects of quality of care [19]. Here we report on the quality of the process of care delivered to small and sick inpatient newborns across diverse facility settings in Nairobi with a view to understanding effective coverage.

## Methods

We conducted a structural assessment and medical record review of neonatal patients admitted between 1 July 2014 and 30 June 2015 to health facilities providing inpatient neonatal care for 24 h a day, seven days a week (24×7) (hereafter referred to as INC facilities), in Nairobi City County, Kenya. All INC facilities were eligible to partake in the study; we explain how facilities were identified in a separate report [19]. Full details of the study protocol have been published elsewhere and all study tools are publicly available [20, 21].

### Study population and sampling

We aimed to provide summary measures of performance for specific indicators of the clinical processes of care across Nairobi City County. To do this, we use proportional sampling (with weighting where this was not achieved) and calculate summary estimates so that each health facility contributes in proportion to its contribution to the total number of annual neonatal admissions across all sites. We estimated that an examination of at least 800 records across the 28 participating sites would be required to meet our aims [19]. The sample of records at each facility was obtained beginning with admissions on 30 June 2015 and moving backwards through admission dates until the desired proportional sample size for that facility was achieved. This strategy was revised for low-volume facilities, for which proportional sampling would lead to <1 record being sampled. For facilities (11 of 28) with 20 or fewer admissions between 1 July 2014 and 30 June 2015, all records of admissions for the entire year were captured. This full record review was also conducted for an additional six facilities that did not have neonatal registers, so we could ascertain the total annual admission numbers for Nairobi.

### Medical record review

Data from neonatal medical records were entered onsite in facilities by trained clerks, following strict standard operating procedures (SOPs), into a purpose-designed standardised data capture tool created in REDCap with inbuilt range and validity checks. Prior to data collection, a mock survey was conducted to pilot the tool, finalise SOPs and train data clerks. During data collection, a 10% random sample of records was independently abstracted by a team supervisor and the two entries were compared. Agreement rates were consistently greater than 95%. Predesigned error-checking scripts were run daily and weekly on collected data and corrections made where possible by referring to source documents.

### Data analysis

Data analysis was conducted using Stata version 13 (Stata Corporation, TX, USA). Correct approaches to care were defined a priori in line with national guidelines that are based on World Health Organization guidelines [22]. Weights were applied during analysis to account for oversampling of records in some facilities. The weighting ensured that each facility contributed to the results as a proportion of their contribution to the total admissions (or sector or size group) across all 28 participating facilities, after exclusion of admissions for supportive care only (e.g. observation after caesarean).

Facilities are presented grouped by sector and size. Three sectors were considered: public, not-for-profit private (mission) and for-profit private (private). Small, medium and large facilities were defined as facilities with <100, 100–900 and >900 admissions between 1 July 2014 and 30 June 2015, respectively. Results by sector and size are presented as pooled patient-level weighted means. Estimates and 95% confidence intervals (CIs) were derived using the svy command in Stata to apply survey weights. MS Excel 2013 was used to produce radar plots, scatter plots and pie charts to present findings on process score domains, heterogeneity of scores across facilities and effective coverage, respectively.

#### Process scores

We assessed six domains of process quality against pre-specified indicators: (I) documentation of newborn characteristics (nine items), (II) documentation of signs and symptoms (16 items), (III) evidence of monitoring (three items), (IV) correct antibiotic dose, (V) correct oxygen treatment and (VI) correct fluids and feeds prescribed (Box 1). For domain IV and domain VI, an overdose and under-dose of antibiotics or fluids and feeds were defined as 20% more or less, respectively, than the recommended dose per kilogram body weight per day in the national guidelines [22, 23]. Unrecorded details of a prescription (e.g. oxygen route) were considered as incorrect prescriptions and included in the denominator when calculating correct prescriptions for domains IV–VI.

Domain scores were calculated at a patient level, as a proportion of the number of correct items within each of the domains I, II and III. In addition, patient-level domain scores (0 = incorrect and 1 = correct) were created for domains IV, V and VI for patients prescribed antibiotics, oxygen or fluids and feeds, respectively. An overall patient-level process score was calculated for each patient by taking the mean of their domain scores. Summary process scores were calculated per facility by taking the mean of all patient process scores within a facility.

#### Structural score

Direct estimation of the overall structural score and the approach taken to data collection have been described elsewhere [19]. Briefly, a structural assessment was conducted in study facilities by clinically trained research team members to examine eight domains in the maternity and neonatal units: (i) infrastructure (three items), (ii) laboratory services (10 items), (iii) hygiene equipment (14 items), (iv) safe delivery equipment and drugs for mothers (37 items), (v) resuscitation equipment for newborns on the delivery ward (20 items), (vi) essential equipment in the newborn unit (NBU) (18 items), (vii) intravenous fluids and feeds in the NBU (eight items) and (viii) NBU drugs (17 items) (listed in Additional file 1: Table S2). An overall structure score was calculated as a percentage from the sum of scores for the eight domains for a facility and divided by the total possible score.

#### Effective coverage

We defined effective coverage as newborns attending a facility providing high-quality care, taking the number of newborns requiring care as the denominator. The gap in receiving INC services and the gaps in quality are calculated as follows.

The gap in receiving INC services was calculated by comparing the number of newborns requiring care to the total number of neonatal admissions to INC facilities recorded in NBU registers during the study period, as previously described [19]. The former was calculated by estimating the number of live births for the 12 months from mid-2014 to mid-2015 in Nairobi City County (120,032) and then we applied a rate of 183 (estimate range 148–221) per 1000 live births requiring inpatient services to this total birth cohort, as also previously described [18]. Thus, the number of newborns requiring care that was used as a denominator when estimating effective coverage was 21,966 (estimate range 17,765–26,527).

Facilities were stratified into high, medium and low quality based on their structural score and summary process score. Facilities with both a summary process score >0.6 and structure score ≥0.8 were considered to be high quality. Newborns attending these facilities were regarded to be receiving effective care. The remaining facilities were stratified as medium quality (summary process score 0.5–0.6 and structure score ≥0.8) or low quality (summary process score ≤0.5 or structure score <0.8). We considered that all newborns admitted to a facility were receiving care equivalent to the level of the facility.

### Ethics and permission

Ethical approval has been granted by the Scientific and Ethics Review Unit of the Kenya Medical Research Institute (KEMRI) (SSC protocol 2999). The study was conducted in collaboration with the Nairobi City County government. Written informed consent to conduct this study was obtained from the medical supervisor or equivalent authority in charge of each facility.

## Results

Of the 34 eligible INC facilities, one was a military hospital with a restrictive access policy and was excluded. Two private facilities (estimated 250–350 maternal deliveries [24] and <50 neonatal admissions each per year) declined to partake in the study and three private facilities (accounting for a combined total of 59 annual neonatal admissions) agreed to the collection of structure data but not to an examination of medical records. We, therefore, report findings from 28 INC facilities: four public hospitals (one medium and three large), six mission hospitals (one small, four medium and one large) and 18 private hospitals (15 small and three large).

After removing admissions for supportive care only (*n* = 98), 1184 admissions of small and sick newborns across the 28 facilities (490 from public, 221 from mission and 473 from private facilities) between 1 July 2014 and 30 June 2015 were sampled from a total of 12,143 admissions.

### Newborn characteristics and outcomes

Newborns were mostly admitted on the first day of life (Table 1). A substantial proportion of newborns weighed <2.5 kg (29.3%). Similarly, a large proportion were preterm (23.2%, <37 weeks gestational age); however, information on gestational age was missing for 39.8% of newborns (Table 1). Admission diagnosis was documented for most (87.0%, 1029/1183) newborns. The most common admission diagnoses were respiratory distress (35.2%), birth asphyxia (32.4%), preterm birth (24.6%), severe infection (18.9%) and jaundice (12.9%). Congenital malformations (3.1%), large for gestational age (3.0%) and dehydration (2.6%) were less common. More than one diagnosis was recorded for 43.8% of newborns.

**Table 1:** Newborn patient characteristics, weighted proportions (%)

	Total (*n* = 1184)	Public (*n* = 490)	Mission (*n* = 221)	Private (*n* = 473)
Age at admission				
<24 h	50.7	48.0	55.2	62.0
1 day	20.0	23.0	15.5	6.3
2–7 days	16.3	17.3	12.3	15.3
>1 week	5.5	5.3	1.7	13.1
Not recorded	7.5	6.4	15.2	3.3
Length of stay				
<7 days	63.8	64.3	81.4	55.0
7–13 days	14.9	18.0	6.3	15.6
14–28 days	7.5	8.4	0.9	9.7
>28 days	4.7	3.9	3.2	6.3
Not recorded	9.1	5.5	8.1	13.3
Sex				
Male	51.2	51.1	50.6	52.8
Female	42.9	42.3	45.4	42.9
Not recorded	5.9	6.6	3.9	4.3
Delivery mode				
Spontaneous	52.4	57.9	44.9	26.1
Caesarean section	40.4	36.7	48.9	52.5
Assisted or breech	1.3	0.7	1.9	4.4
Not recorded	6.0	4.8	4.3	17.0
Birthweight				
<1 kg	1.2	0.8	0.6	4.8
1–1.4 kg	6.2	6.5	2.1	10.9
1.5–2.4 kg	21.9	23.4	13.7	24.3
2.5–4 kg	58.8	59.7	67.5	39.2
>4 kg	5.4	4.6	8.9	5.4
Not recorded	6.4	4.9	7.2	15.3
Gestational age				
Very preterm (<32 wk)	7.0	7.0	2.2	14.6
Moderate or late preterm (32–36 wk)	16.2	15.5	13.3	25.4
Term (37–42 wk)	37.0	44.3	13.2	24.5
Not recorded	39.8	33.1	71.3	35.5
Apgar at 5 min				
0–3	2.2	2.9	0.6	0.2
4–6	14.6	15.9	12.9	8.2
7–10	69.3	69.6	74.9	58.9
Not recorded	13.8	11.6	11.5	32.8
HIV exposure status				
Exposed	4.4	5.0	3.2	2.6
Not exposed	58.2	66.5	41.6	27.1
Not recorded	37.3	28.5	55.2	70.4

Weighted proportions ensure that each facility contributed to the results as a proportion of their contribution to the total admissions across facilities and within their sector. The total refers to weighted results across all 28 facilities

Outcome was missing for 3.0% of newborns. Where recorded (*n* = 1104), 90.6% were discharged alive, 7.2% died, 1.7% were referred and 0.5% absconded. Crude mortality, without adjustment for case-mix or acuity, was higher in public facilities (8.8% [95% CI: 6.5–11.7%]) compared with mission facilities (2.1% [95% CI: 0.3–12.3%]) and private-sector facilities (3.8% [95% CI: 2.2–6.5%]).

### Quality of process of care

**Fig. 1:**
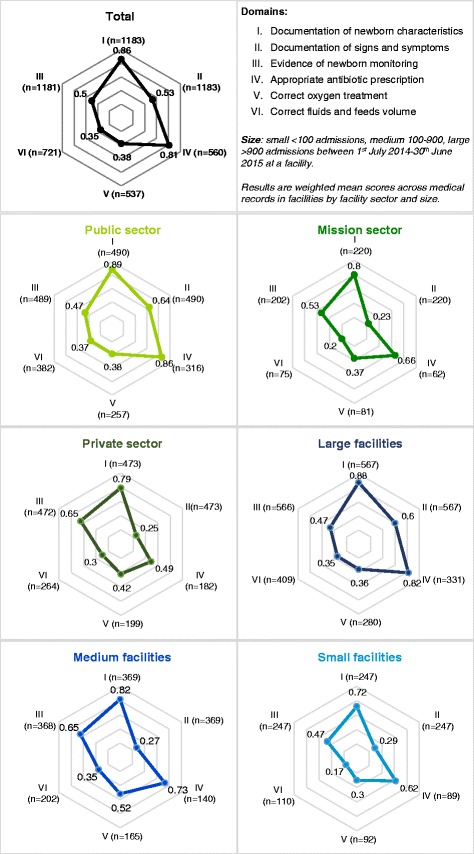
Quality of care domain scores by sector and size of facilities

Figure 1 presents radar plots summarising domain scores for Nairobi City County as a whole (Fig. 1a), by sector (Fig. 1b, c and d) and facility size (Fig. 1e, f and g). Mean (95% CI) scores are presented in Additional file 1: Table S3. Overall, performance was lowest for domain VI, correct fluid and feed volume (weighted mean score 0.35 [95% CI: 0.22–0.48]); whereas, performance was best for domain I, documentation of newborn characteristics (0.86 [95% CI: 0.80–0.93]).

**Fig. 2:**
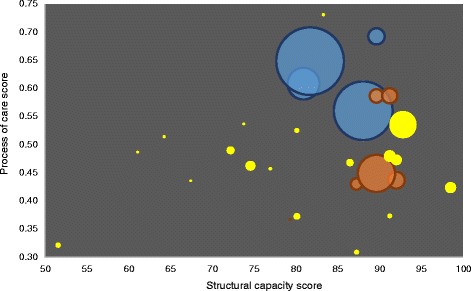
Relationship between summary process of care score and structural capacity score for each facility, by patient volume and sector. Each bubble represents a facility. Bubble sizes are proportional to the number of admissions received by the facility between 1 July 2014 and 30 June 2015. Sectors are represented by bubble colours: blue = public, mission = orange and private = yellow. Scores are summary scores for patient records within each facility. Note that the *x*- and *y*-axes do not begin at zero

When stratifying by sector and size, different patterns emerge, with generally lower domain scores for the process of care in the private (summary process score = 0.49 [95% CI: 0.44–0.54]) and mission sectors (0.48 [95% CI: 0.43–0.53]) compared with public-sector facilities (0.61 [95% CI: 0.56–0.67]), which account for 71% of admissions. Large facilities (three public, one mission and three private facilities) tended to have higher domain scores (summary process score = 0.59 [95% CI: 0.52–0.66]), while medium and small facilities scored less well (0.54 [95% CI: 0.48–0.60] and 0.45 [95% CI: 0.42–0.48], respectively), with suggestions of a trend of improving scores from small to larger facilities (Fig. 2).

#### Domain-specific indicators

Specific and additional details of domain indicators are presented in Table 2. Of note for domains I and II, documentation in the public sector was slightly better than in the private and mission sectors, particularly for documentation of key signs and symptoms.

**Table 2:** Documented newborn assessment, monitoring and clinical care in newborn medical records, weighted proportions (%, 95% CI)

	Total	Public	Mission	Private
Documentation of newborn characteristics	*n* = 1182	*n* = 490	*n* = 220	*n* = 472
Characteristics documented (of nine), mean (95% CI)	7.8 (7.2–8.3)	8.0 (7.3–8.7)	7.2 (6.9–7.5)	7.1 (6.4–7.7)
Documentation of signs and symptoms	*n* = 1182	*n* = 490	*n* = 220	*n* = 472
Signs documented (of eight), mean (95% CI)	4.3 (2.7–5.9)	5.2 (4.0–6.5)	1.7 (0.8–2.5)	2.1 (1.7–2.5)
Symptoms documented (of eight), mean (95% CI)	4.2 (2.2–6.2)	5.0 (2.9–7.2)	2.0 (1.5–2.6)	1.9 (1.3–2.6)
Evidence of patient monitoring	*n* = 1182	*n* = 490	*n* = 220	*n* = 472
Evidence of further clinical review	89.6 (85.5–92.6)	90.7 (87.2–93.3)	83.6 (77.9–88.0)	91.6 (72.2–97.9)
Frequency of temperature recorded in first 48 h, mean (95% CI)	6.6 (5.0–8.2)	5.2 (4.5–5.8)	6.9 (5.6–8.2)	9.6 (8.2–10.9)
Treatment sheet available and filled	88.7 (79.9–93.9)	91.0 (81.7–95.9)	80.0 (54.2–93.1)	86.3 (76.8–92.2)
Vital signs chart available and filled	39.4 (13.3–73.4)	26.6 (4.7–72.8)	72.9 (48.8–88.3)	75.2 (39.0–93.5)
Feeding chart available and filled*	35.7 (23.3–50.3)	31.7 (20.7–45.1)	30.4 (9.6–64.3)	81.5 (48.3–95.4)
Fluid chart available and filled**	41.4 (17.7–70.0)	35.1 (9.8–72.8)	42.2 (23.9–62.9)	79.8 (40.9–95.8)
Weight monitored and filled	21.3 (7.8–46.3)	22.9 (6.2–57.1)	5.9 (3.0–11.2)	34.8 (14.6–62.6)
Appropriate antibiotic prescription				
Antibiotic dose	*n* = 556	*n* = 316	*n* = 62	*n* = 178
Incorrect dose	19.4 (11.8–30.2)	14.4 (8.6–23.1)	34.2 (15.9–58.7)	51.0 (38.6–63.4)
Gentamicin doseǂ	*n* = 539	*n* = 315	*n* = 62	*n* = 162
Overdose	11.7 (7.1–18.5)	9.4 (5.5–15.5)	11.6 (7.4–17.8)	36.0 (26.0–47.3)
Under-dose	4.6 (2.3–9.1)	2.9 (1.6–5.5)	15.4 (7.0–30.8)	8.5 (6.0–11.8)
Penicillin doseǂ	*n* = 512	*n* = 302	*n* = 43	*n* = 167
Overdose	2.8 (1.4–5.3)	2.7 (1.2–5.8)	0.0 (0.0–0.0)	5.8 (4.0–8.5)
Under-dose	3.6 (1.2–10.6)	1.3 (0.8–2.2)	21.6 (2.5–74.8)	11.1 (8.1–14.9)
Correct oxygen treatment				
Those requiring and prescribed	60.8 (54.8–66.5) (*n* = 316)	59.2 (53.6–64.6) (*n* = 187)	70.8 (42.0–89.0) (*n* = 26)	69.0 (53.9–80.9) (*n* = 103)
Correct route (nasal prongs or catheter)	36.4 (24.6–50.0) (*n* = 412)	36.5 (21.5–54.7) (*n* = 181)	35.4 (16.9–59.5) (*n* = 73)	37.0 (27.5–47.6) (*n* = 158)
Correctly prescribed (required and correct route)	40.7 (23.7–60.4) (*n* = 191)	39.4 (19.7–63.2) (*n* = 111)	57.1 (39.8–72.8) (*n* = 18)	39.1 (26.6–53.3) (*n* = 62)
Correct fluids and feeds	*n* = 721	*n* = 382	*n* = 75	*n* = 264
Correct fluid and feed volumes	34.9 (23.2–48.7)	37.4 (23.6–53.6)	19.9 (5.5–51.6)	30.1 (20.0–42.6)

Overdose and under-dose was defined as 20% more and less, respectively, than the recommended dose per kilogram body weight per day as per national guidelines. Correct fluid and feed volume allow for 20% margin of error from national guidelines

*Of those patients who received a prescription for feeding (*n*: total = 307, public = 199, mission = 16 and private = 92)

**Of those patients who received a prescription for fluids (*n*: total = 529, public = 252, mission = 66 and private = 211)

ǂ Of those patients who received antibiotics (*n* = 640) and had their weight, prescription frequency and dose recorded

CI confidence interval

Most newborns had evidence of a further clinical review after admission and vital signs monitoring within the first 48 h after admission. However, vital signs were predominantly recorded only in nursing notes (to which clinicians rarely have access). Only 39.4% of newborns had a vital signs chart available and completed in the medical record (Table 2). Similar proportions of newborns had feeding charts and fluids charts available and filled. The level of evidence for weight monitoring was particularly low (21.3%).

Almost two-thirds (63.4%) of the population were prescribed antibiotics, of whom the majority (92.6%) were prescribed both gentamicin and penicillin, suggesting good adherence to recommended first-line treatment across sectors. A large proportion (62.5%) of newborns who had no admission diagnosis of severe infection (*n* = 821) were nonetheless prescribed antibiotics. Incorrect doses were prescribed to 19.4% of newborns. An overdose of gentamicin was the most common error (11.7%) (Table 2).

Of those prescribed oxygen, 36.4% were prescribed oxygen via the correct route (nasal prongs or catheter); 32.5% had no route prescribed and 23.5% were prescribed oxygen via a mask. Recorded signs and symptoms suggested 39.7% (*n* = 894) might have required oxygen; 60.8% (*n* = 316) of these newborns had an oxygen prescription documented, of whom 40.7% (*n* = 191) were also prescribed the correct route.

Feeds were prescribed to 33.6% of newborns and 51.1% were prescribed fluids. Of those prescribed either feed or fluid (*n* = 721), 17.8% were prescribed both. Only 34.9% of newborns were found to be prescribed the correct volume of feed or fluid based on their admission age and weight (Table 2).

### Structural and process quality

The weighted mean overall process of care score was 0.58 (95% CI: 0.52–0.64) across newborn records (Additional file 1: Table S3). The score was higher among public (0.61, 95% CI: 0.56–0.67), compared with mission (0.48, 95% CI: 0.43–0.53) and private (0.49, 95% CI: 0.44–0.54) facilities.

Visual assessment of Fig. 2 suggests a possible relationship between facility size and summary process and structural capacity scores, with larger facilities tending towards higher scores and smaller facilities tending towards lower scores for both process and structure. Large heterogeneity in process scores exists among facilities with high structural capacity scores.

Facilities with an absence of dedicated nursing staff on the NBU (associated with small facility size) had a lower mean process score than where facilities had nurses specifically assigned to NBUs (mean scores 0.47 [95% CI: 0.43–0.51], *n* = 410 and 0.60 [95% CI: 0.54–0.65], *n* = 774, respectively).

### Effective coverage

Of newborns receiving services at one of the 28 study facilities, we estimate that 45.0% (5459/12,143) are receiving care associated with high to medium summary process scores (>0.6), while 35.4% (4300/12,143) and 19.6% (2384/12,143) are receiving care associated with medium to low (0.5–0.6) and low summary process quality scores (≤0.5), respectively.

**Fig. 3:**
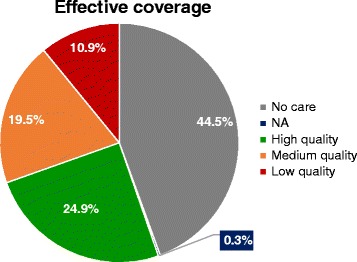
Levels of care environment for newborns requiring inpatient services (*n* = 21,966) in Nairobi City County. No care: Not attending one of the 31 INC facilities. NA: Attended one of the 31 inpatient neonatal care facilities but did not partake in process assessment. High quality: Facility process score >0.6 and structure score ≥0.8. Medium quality: Facility process score 0.5–0.6 and structure score ≥0.8. Low quality: Facility process score ≤0.5 or structure score <0.8

We estimate that 24.9% [5459/21,966; estimate range 20.6% (5459/17,765) to 30.7% (5459/26,527)] of newborns needing care are admitted to a facility that provides high-quality care (process score >0.6 and structure score ≥80%), and are, hence, receiving effective coverage (Fig. 3). A further 30.4% of newborns needing care are receiving services either in a medium-quality care environment (19.5%, process score 0.5–0.6 and structure score ≥ 80%) or a low-quality care environment (10.9%, process score ≤ 0.5 or structural score <80%). Previous reports [18] estimated that 44.5% [9764/21,966; estimated range 37% (9764/17,765) to 55% (9764/26,527)] of newborns requiring care do not attend an INC facility in Nairobi City County and we regard these newborns as not receiving care. A further 0.3% (59/21,966) receive services at the remaining INC facilities not included in this process of care assessment.

## Discussion

Our study examines the quality of neonatal care across an entire population, capturing information across sectors, and working towards defining effective coverage [25]. Our results provide insight into key actionable quality gaps and unearthed large heterogeneity in care contexts and quality for neonatal patients in this high-mortality setting. Deficits were most commonly identified in small, often private facilities, although severe workforce deficits and overcrowding undermine the quality of care in the public sector. Taken together with results of linked studies, we estimate that effective service provision is unavailable for 75% of sick newborns in Nairobi City County, this gap being made up of failures in access (44.5%) and receipt of lower quality services (30.4%).

Documentation of basic newborn characteristics, diagnoses and outcomes are important sources of data for local statistics to inform public health and health-care planning. As others have also found, improvement in the documentation of patient characteristics and care is needed, especially in mission and private-sector facilities [11, 13]. Standard admission forms that are aligned with national guidelines, used more often in public-sector facilities, may help to improve documentation [26, 27]. Structured vital signs charts were found to be commonly available but only completed in a third of records. Instead, newborn monitoring was often documented in the unstructured nursing Kardex (nursing-specific notes), to which clinicians do not have access. Combined medical and nursing records (a suggestion made over 60 years ago [28]) might, therefore, be a useful intervention to improve documentation and clinical communication.

Documented clinical care that does not align with national guidelines was common. Of particular concern is the possible harm caused by the inappropriate prescription of antibiotics, one of five indicators of quality neonatal care recommended by international and national experts [23]. In our study, antibiotics were prescribed to the majority (63.4%) of newborns, regardless of a diagnosis of sepsis. Encouragingly though, first-line antibiotic regimens were used in over 90% of cases. Despite this, consistent with previous findings [13], antibiotic doses that were 20% too high or too low were prescribed for more than a fifth of newborns. Overdosing of gentamicin, which may cause toxicity in newborns, was of most concern. Mitigating this risk, however, the length of stay of newborns was often short (median = 4 days, interquartile range = 2–7.6, *unpublished data*). Nonetheless, inaccurate prescribing of a potentially toxic drug is an important patient safety issue. Also of concern are the prescribing inaccuracies in 65% of newborns receiving feeds or fluids. As with inaccurate drug prescription, this was more common in smaller facilities.

A distinct group of small facilities were found to have very low structural and process quality. This clustering of deficits was found primarily among private facilities, specifically smaller private facilities. Though highly heterogeneous as a sector, private facilities are least likely to be involved in training and quality improvement activities initiated by the government or partners. Minimum standards should be ensured across all settings and sectors, perhaps through benchmarking, accreditation and external audits. In some settings, this has been achieved through voluntary networks across public and private facilities [29].

Until recently, neonatal health care was not prominent on the global or national agendas, limiting investment in services for this patient group. Although providing good quality neonatal care poses specific challenges, many of the health system’s weaknesses experienced in low-resource settings are also evident in Nairobi, impacting on neonatal survival. These weaknesses included health worker shortages in the public sector, poor staff motivation and inadequate health-care financing more broadly (as evidenced by the recent doctors’ and nurses’ strikes over pay and working conditions). The specific reasons behind gaps in quality of care for neonates and the challenges faced by nurses working in NBUs are being examined through ongoing qualitative research in Nairobi.

There are strengths and limitations to our study. Our use of routine medical records for the evaluation of quality of care is a pragmatic approach that can be integrated into routine on-site quality assessment in facilities [26, 30]. However, it also limits assessment to indicators that are routinely documented. Furthermore, medical records are not standardised across facilities, which may have contributed to the lower performance in non-public-sector facilities. We defined correct care a priori based on national standards of care, which have been widely disseminated in Kenya [22]. However, such guidelines may have less penetration in the private sector and, in particular, large private hospitals may have their own standards of care drawn from high-income practice settings. This may slightly disadvantage large private facilities in our scoring approach. Due to the lack of adequate data on acuity and case mix, we were unable to evaluate links between the quality scores and mortality meaningfully. We took a non-randomised approach to sampling records where this was needed (11/28), focusing on the most recent admissions. We do not anticipate that this should bias our findings unless seasonality differences in quality of care exist. Our aim was to provide insight into the entire population of neonatal patients receiving 24×7 inpatient care in Nairobi City County. Four of the 32 eligible facilities declined to partake. However, it is estimated that their contribution to annual admissions was a maximum of 1.3% (159/12,302) and, hence, we do not anticipate that our overall results would change upon their inclusion. Our denominator for effective coverage relies on estimating the number of live births in Nairobi City County during the study period and the proportion of those live births requiring admission. Although we have provided uncertainty estimates for the latter, we are unable to quantify uncertainty around the former estimate. The true range of uncertainty around our estimate for effective coverage is, therefore, higher than presented, which should be taken into consideration when interpreting these results. A detailed description of the methods and limitations of calculating the coverage denominator are presented in a separate publication [19].

## Conclusions

Effective coverage of interventions delivered at facility level will become increasingly important for reducing neonatal mortality. Our multi-sector landscape evaluation of almost all facilities providing INC services to small and sick newborns in Nairobi City County demonstrates the value of estimating effective coverage and identifying gaps in access and quality. To improve these estimates continuously and ensure progress in addressing gaps can be tracked, it is important to develop the capacity to monitor access and quality across all sectors routinely. Efforts to improve care would be facilitated by the development and implementation of common minimum standards for facilities and processes of care, including the standardisation of medical and nursing records. Strategies to improve facility-based care for newborns will need to consider substantial deficits in access, resources and processes of care as part of long-term planning to improve service delivery equitably.

## Box 1: Process score domains

**Domain I: Documentation of newborn characteristics**

**• Characteristics:** Age, sex, mode of delivery, weight, gestational age, Apgar score at 5 min, HIV status, diagnosis, outcome

**•** Score of 0–1 as a proportion of 0–9 characteristics among all patients

**Domain II: Documentation of signs and symptoms (documentation of absence or presence)**

**• Signs** (evaluation on admission): Temperature, bulging fontanelle, can suck or breastfeed, reduced mobility or floppy, respiratory rate, indrawing, grunting, central cyanosis

**• Symptoms** (history): Prolonged rupture of membranes (ROM) (>18 h), fever, difficulty breathing, severe vomiting, difficulty feeding or breastfeeding, convulsions, partial or focal fits, apnoea

**•** Score of 0–1 as a proportion of 0–16 signs and symptoms among all patients

**Domain III: Evidence of monitoring**

**• Evidence:** Treatment sheet available and filled, vital signs chart available and filled, evidence of weight monitoring

**•** Score of 0–1 as a proportion of 0–3 monitoring evidences among all patients

**Domain IV: Appropriate antibiotic prescription**

**• Dose** of gentamicin and/or penicillin as per national guidelines, allowing for ±20% margin of error

**•** Score of 1 for correct dose and 0 for incorrect dose among patients with prescription for gentamicin or penicillin

**Domain V: Correct oxygen prescription**

**• Correct oxygen prescription:** Correct route and prescribed to patients requiring oxygen treatment as per recorded signs and symptoms

**•** Score of 1 for both components correct, 0.5 for one component correct and 0 for neither correct among patients prescribed oxygen treatment

**Domain VI: Correct fluids and feeds volume**

**• Volume** as per national guidelines, allowing for ±20% margin of error

**•** Score 1 for correct volume and 0 for incorrect volume among patients with fluid or feed prescription

## Additional file

Additional file 1: **Table S1.** Domains of process score. **Table S2.** Domains of structural score. **Figure S1.** Percentage of signs and symptoms documented in newborn medical records. **Table S3.** Domain scores by sector and size of facilities. (DOCX 32 kb)

## Abbreviations

CI: Confidence interval
INC: Inpatient neonatal care
KEMRI: Kenya Medical Research Institute
NBU: Newborn unit
SOP: Standard operating procedure
