# Supplementary material for: Effective coverage of essential inpatient care for small and sick newborns in a high mortality urban setting: a cross-sectional study in Nairobi City County, Kenya
Source: BMC Med. 2018 May 22;16:72. doi: 10.1186/s12916-018-1056-0 (PMC5963150; doi:10.1186/s12916-018-1056-0)
Supplement: Supplementary file 1 — Table S1. Domains of process score. Table S2. Domains of structural score. Figure S1. Percentage of signs and symptoms documented in newborn medical records. Table S3. Domain scores by sector and size of facilities. (DOCX 32 kb) [file 12916_2018_1056_MOESM1_ESM.docx]

**Table S1: Domains of process score**

| **Domain** | **Patient group/ denominator** | **Components** | **Score** |
| --- | --- | --- | --- |
| (i) Documentation of newborn characteristics | All patients | 1. Age 2. Sex 3. Mode of delivery 4. Weight 5. Gestational age 6. APGAR score at 5 minutes 7. HIV status 8. Diagnosis 9. Outcome | 0-1 as a proportion of 0-9 |
| (ii) Documentation of signs and symptoms (documentation of absence or presence) | All patients | Signs (evaluation on admission):   1. Temperature 2. Bulging fontelle 3. Can suck/breastfeed 4. Reduced mobility/floppy 5. Respiratory rate 6. Indrawing 7. Grunting 8. Central syanosis   Symptoms (history):   1. Prolonged ROM (>18 hours) 2. Fever 3. Difficulty breathing 4. Severe vomiting 5. Difficulty feeding/breastfeeding 6. Convulsions 7. Partial/focal fits 8. Apnoea | 0-1 as a proportion of 0-16 |
| (iii) Evidence of monitoring | All patients | 1. Treatment sheet available and filled 2. Vital signs chart available and filled 3. Evidence of weight monitoring | 0-1 as a proportion of 0-3 |
| (iv) Appropriate antibiotic prescription | Patients with prescription for gentamicin or penicillin | Dose of gentamicin and/or penicillin as per national guidelines, allowing for +/- 20% margin of error. | 0: incorrect dose  1: correct dose |
| (v) Correct oxygen prescription | Patients with prescription for oxygen treatment | 1. Correct route 2. Required oxygen treatment as per recorded signs and symptoms | 0: neither correct  0.5: one component correct  1: both components correct |
| (vi) Correct fluids and feeds volume | Patients with fluid and/or feed prescription | Volume as per national guidelines, allowing for +/- 20% margin of error. | 0: incorrect volume  1: correct volume |

**Table S2: Domains of structural score**

| **Domain** | **Components** | **Score** |
| --- | --- | --- |
| (i) Infrastructure | 1. Consistent power (outages <monthly) or generator serving NBU 2. Heating in NBU 3. Running water | 0-1 as a proportion of 3. |
| (ii) Laboratory services | 1. Minimum package of care: 2. Open 24/7 3. Test for haemoglobin 4. Test for bilirubin (blood test) 5. Glucose tests 6. Blood grouping and cross match 7. Electrolytes (sodium / potassium) 8. Blood bank 9. Blood slide microscopy for malaria parasites 10. Test for direct Coombs test 11. Urea or creatinine 12. Liver function tests (enzymes e.g. AST/ALT) 13. Microscopy & culture: Pus swab and urine culture 14. CSF microscopy 15. Coagulation profile 16. Blood culture ability | Minimum package of care required, otherwise score=0.  0-1 as a proportion of 10 components. |
| (iii) Hygiene (on delivery ward and newborn unit) | 1. Cleaning/disinfectant supplies 2. Sharps disposed in a special container 3. Clean gloves available 4. Separate clinical and non-clinical waste 5. Sinks with soap and water for hand-washing 6. Mother has access to running water 7. Alcohol hand rub | 0-1 as a proportion of 14 (7 for delivery ward + 7 for NBU).  0-1 as a proportion of 7 in facilities without a maternity unit |
| (iv) Safe delivery equipment and drugs for mother | **Equipment**   1. Thermometer 2. Sterile syringes 3. Sterile needles 4. Sterile vaginal examination packs 5. Sterile delivery set (complete) 6. A stethoscope 7. Amnicots/sterile Kocker’s forceps for artificial rupture of membrane 8. Urine dipstick kits/strips 9. Urinary catheters 10. Vacuum (such as Kiwi) for assisted vaginal delivery 11. Manual vacuum aspirator (MVA) 12. Long gloves for manual removal of placenta 13. Guedel airways – these should be a full range of sizes 14. Bag Valve Mask (BVM) device: adult size bag and mask 15. Oxygen source (any and working) 16. Nasal catheters/prongs 17. Oxygen face –masks (with and without reservoir bags) 18. Oxygen flow regulators 19. Laryngoscope 20. Laryngoscope blades (straight, curved, and different sizes) 21. Endotracheal tubes (of different sizes) 22. IV fluid giving sets 23. Blood giving set 24. Adult IV cannulae 25. Blood pressure monitor (any and working)   **Drugs**   1. Adrenaline 2. Magnesium sulphate 3. Lasix 4. Digoxin 5. Morphine 6. Oxytocin 7. Dexamethasone 8. Prostaglandin F2 alpha 9. Calcium gluconate 10. Penicillin 11. Gentamicin 12. Ceftriaxone/Cefuroxime | Score only applies to facilities with a maternity unit.  0-1 as a proportion of 37. |
| (v) Neonatal resuscitation equipment | 1. Thermometer 2. Weighing scales 3. Sterile syringes 4. Sterile needles 5. Warm dry towels for dying and wrapping the newborn 6. Sterile cord clamp 7. Sterile scissors 8. A firm stable surface for placing the newborn for resuscitation (where warmth can be maintained) 9. An overhead light source above the surface for resuscitation 10. A clock in view or reach of surface for resuscitation 11. A stethoscope 12. Suction tubes/catheters 13. Suction Machine 14. Guedel airways – these should be a full range of sizes 15. Bag Valve Mask (BVM) devices: bag size 500 ml or750 ml, that are in working order with newborn face masks (sizes 0 and 1) 16. Oxygen source (any and working) 17. Nasal catheters/prongs 18. Oxygen face –masks (with and without reservoir bags) 19. Oxygen flow regulators 20. Warming equipment-working radiant heaters | Score only applies to facilities with a maternity unit.  0-1 as a proportion of 20. |
| (vi) Essential ward equipment in the NBU for treatment and diagnostic procedures | 1. Thermometer 2. Weighing scales 3. Sterile syringes 4. Sterile needles 5. A stethoscope 6. Suction tubes/catheters 7. Suction Machine 8. Guedel airways – these should be a full range of sizes 9. Bag Valve Mask (BVM) devices: bag size 500 ml or750 ml, that are in working order with newborn face masks (sizes 0 and 1) 10. Oxygen source (any and working) 11. Nasal catheters/prongs 12. Oxygen face –masks (with and without reservoir bags) 13. Oxygen flow regulators 14. Warming equipment-working radiant heaters 15. Kangaroo mother care wraps 16. Phototherapy equipment 17. Eye protection for phototherapy 18. Blood transfusion giving set | 0-1 as a proportion of 18. |
| (vii) IV fluid and feeds in the NBU | 1. Feeding cups for giving expressed breast milk 2. IV fluid burette 3. Infusion set / adult IV fluid set 4. Paediatric cannulae 5. Nasogastric tube (FG6 or 8 or other) 6. glucose 10% 7. normal saline IV or ringers lactate 8. term formula | 0-1 as a proportion of 8. |
| (viii) NBU drugs | 1. Vitamin K 2. Nevirapine solution 3. Prophylactic tetracycline eye ointment* 4. Phenobarbitone (injection) 5. Phenytoin (injection) * 6. Aminophyline* 7. Penicillin (injection) 8. Gentamicin or Amikacin 9. Ampicillin / Cloxacillin (injection) * 10. Oral Cloxacillin * 11. Oral erythromycin* 12. Metronidazole (injection) * 13. Ceftriaxone or cefotaxime* 14. Ferrous Fumarate suspension* 15. Folate drops* 16. Multivitamin syrup/drops* 17. Intravenous (Anti-D) immunoglobulin (for rhesus disease) * | 0-1 as a proportion of 17. |

** Considered available if on the ward or in a store*

*Equipment was considered to be available if it was both on the ward and working on the day of the visit. Items available on either the maternity ward or NBU that were described by facility staff as being shared between the two locations, were considered to be available for both maternity and the NBU.*

*Drugs were considered to be available if they were on the ward or accessible within five minutes without administrative barriers (such as prepayment or waiting for keys from a senior hospital staff member not present on the ward). A drug was considered to be ‘in store’ if it was available within the facility and within 2 hours of request but not available on the ward (by the above criteria).*

**Figure S1:** Percentage of signs and symptoms documented in newborn medical records.

|  | **% Documented (as either absent or present)** | | | | |
| --- | --- | --- | --- | --- | --- |
| **Symptoms** | Total | Public | Mission | Private |  |
| Difficulty breathing | **100** | **100** | **100** | **100** |  |
| Difficulty feeding | **54.1** | **66.2** | **23.2** | **19.9** |  |
| Fever | **49.8** | **61.8** | **16.3** | **19.9** |  |
| Vomits everything | **49.3** | **62.6** | **13.5** | **14.0** |  |
| Convulsions | **50.1** | **64.2** | **13.5** | **11.0** |  |
| Apnoea | **48.9** | **62.4** | **13.0** | **11.9** |  |
| Partial/focal fits | **48.4** | **62.6** | **12.9** | **6.5** |  |
| PROM | **19.2** | **22.1** | **12.2** | **10.4** |  |
|  |  |  |  |  |  |
| **Signs** |  |  |  |  |  |
| Cyanosis | **66.9** | **83.7** | **20.5** | **23.8** |  |
| Severe in-drawing | **62.5** | **78.4** | **14.2** | **29.3** |  |
| Muscle tone | **57.4** | **70.8** | **21.2** | **21.6** |  |
| Bulging fontanelle | **57.8** | **70.1** | **18.4** | **35.0** |  |
| Grunting | **55.9** | **68.8** | **17.7** | **27.4** |  |
| Suck reflex/ability to feed | **51.1** | **61.9** | **21.3** | **23.6** |  |
| Respiratory rate | **47.6** | **56.3** | **22.8** | **26.6** |  |
| Temperature | **32.1** | **33.9** | **30.6** | **22.2** |  |

**Table S3:** Domain scores by sector and size of facilities.

| **Domain** | **Overall**  **(n=1184)** | **Sector** | | | **Size** | | |
| --- | --- | --- | --- | --- | --- | --- | --- |
|  |  | **Public**  **(n=489)** | **Mission**  **(n=220)** | **Private**  **(n=472)** | **Small**  **(n=247)** | **Medium**  **(n=370)** | **Large**  **(n=567)** |
|  | **Weighted mean (95% CI) scores 0-1** | | | | | | |
| Documentation of newborn characteristics | 0.86 (0.80-0.93) | 0.89 (0.81-0.97) | 0.80 (0.76-0.83) | 0.79 (0.72-0.86) | 0.72 (0.66-0.79) | 0.82 (0.76-0.87) | 0.88 (0.80-0.95) |
| Documentation of signs and symptoms | 0.53 (0.31-0.75) | 0.64 (0.43-0.85) | 0.23 (0.16-0.30) | 0.25 (0.19-0.32) | 0.29 (0.23-0.35) | 0.27 (0.19-0.34) | 0.60 (0.36-0.83) |
| Appropriate antibiotic prescription | 0.81 (0.71-0.90) | 0.86 (0.78-0.93) | 0.66 (0.43-0.88) | 0.49 (0.36-0.62) | 0.62 (0.56-0.68) | 0.73 (0.45-1.01) | 0.82 (0.74-0.91) |
| Correct oxygen prescription | 0.39 (0.33-0.44) | 0.38 (0.33-0.44) | 0.37 (0.12-0.61) | 0.42 (0.32-0.51) | 0.30 (0.18-0.42) | 0.52 (0.44-0.61) | 0.36 (0.29-0.43) |
| Correct feed and fluids volume | 0.35 (0.22-0.48) | 0.37 (0.22-0.53) | 0.20 (-0.03-0.43) | 0.30 (0.19-0.42) | 0.17 (0.08-0.25) | 0.35 (0.29-0.41) | 0.35 (0.20-0.51) |
| Evidence of newborn monitoring | 0.50 (0.34-0.65) | 0.47 (0.27-0.66) | 0.53 (0.42-0.64) | 0.65 (0.45-0.86) | 0.47 (0.36-0.59) | 0.65 (0.45-0.85) | 0.47 (0.29-0.64) |
| **Total process score** | **0.58 (0.52-0.64)** | **0.61 (0.56-0.67)** | **0.48 (0.43-0.53)** | **0.49 (0.44-0.54)** | **0.45 (0.42-0.48)** | **0.54 (0.48-0.60)** | **0.59 (0.52-0.66)** |

*Size: small <100 admissions, medium 100-900, large >900 admissions between 1st July 2014 and 30th June 2015 at a facility.*
